# The mesenchymal stem cell secretome as an acellular regenerative therapy for liver disease

**DOI:** 10.1007/s00535-019-01599-1

**Published:** 2019-07-03

**Authors:** Julia Driscoll, Tushar Patel

**Affiliations:** Department of Transplantation, Mayo Clinic, 4500 San Pablo Road, Jacksonville, FL USA

**Keywords:** Extracellular vesicles, Secretome product, Stem cells, Regenerative medicine

## Abstract

The use of mesenchymal stem cells (MSC) for tissue repair has garnered much interest and has been evaluated in several disease settings. Recent evidence indicates that the beneficial effects observed with MSC-based therapy can be mediated through the paracrine release of extracellular vesicles and other soluble proteins or biologically active molecules, which collectively constitute the MSC secretome. In this concise overview, we highlight results from preclinical and other studies that demonstrate the therapeutic efficacy of the MSC secretome for diseases that are characterized by liver injury or fibrosis. The potential for the use of the MSC secretome as an acellular regenerative therapy and approaches for the isolation of a secretome product for therapeutic applications are highlighted. The use of the MSC secretome as an acellular therapeutic agent could provide several advantages over the use of cell-based therapies for liver diseases.

## Introduction

An underlying pathophysiological feature in many diseases affecting the liver, irrespective of the etiology, is the presence of hepatic injury and inflammation. Acute liver injury can occur as a consequence of ischemic, toxic–metabolic, cytotoxic or other insults. Exposure to such insults can elicit tissue inflammation as well as result in innate and adaptive immune responses that could also directly contribute to further injury. If the injury or inflammation persists and the responses are unchecked, hepatic fibrosis can develop. The accumulation of fibrotic tissue may not be fully reversible, and can be progressive. In the absence of any effective therapies to ameliorate or reverse fibrotic changes, the only therapy currently available to those with advanced chronic liver disease is hepatic replacement with a liver transplant. Therapies that can repair or regenerate the liver in the setting of persistent injury or inflammation are needed to avoid reliance on replacement therapies such as transplantation which are not a viable option for many patients.

## Mesenchymal stem cells (MSC) in liver injury

Regenerative therapies involving the use of MSC are a promising therapeutic approach for reducing liver injury, modulating the immune response to injury, and enhancing repair and regeneration of hepatic epithelia. MSC are cells with differentiation capability which were first isolated from bone marrow but can be derived from perivascular cells from a number of tissues, including the liver. Liver MSC are elongated and spindle-shaped, and express stem-cell markers such as vimentin and MSC markers such as CD90, but not markers of hematopoietic stem cells such as CD45, or markers of other liver progenitor cells such as CK19 [1]. MSC from sites other than the liver such as the bone marrow can also be recruited to the liver when there is injury present.

The secretome from MSC that are resident in the liver or MSC that are recruited to the liver could have functional effects [2], [3]. MSC have several characteristics that contribute to their reparative and regenerative properties. First, MSC have the capacity for multi-lineage differentiation into a myriad of different cell types. Next, they have migratory and homing capabilities that enable their sequestration into regions of injury. Their capacity for diapedesis across the endothelium is enabled by the cell surface expression of chemokine receptors, adhesion antigens that ensure cell binding to the endothelium wall, and the expression and subsequent release of matrix metalloproteases (MMP) and other proteolytic enzymes [4]. Furthermore, through the release of anti-inflammatory cytokines and factors, MSC have immunomodulatory effects on both the innate and adaptive immune systems ([5],[6, 7],^8^]). Moreover, MSC are capable of releasing proteins and extracellular vesicles (EV) that have been shown to directly modulate liver injury in different models.

Despite their beneficial properties, there are several limitations to the use of MSC as cellular therapies. These include the potential for aberrant differentiation, tumor formation [9] and low engraftment [10]. The underlying concerns of the potential for tumor formation or differentiation into undesirable cell types have hindered the adoption and use of MSC-based therapeutic approaches, even though these risks remain unsubstantiated [11]. The half-life of transplanted MSC may be inadequate for tissue regeneration by MSC differentiation. Systemically delivered allogeneic MSC tend to accumulate in the lungs within the first 24 h of transplant; those that escape entrapment by the lungs sequester within the liver and spleen [12, 13]. Elimination by adaptive immune cells and the loss of their immune privileged status can both contribute to the short half-life of transplanted MSC [13]. Allogeneic MSC that engraft within target organs can lose their immune privileged status due to the surface expression of major histocompatibility complex class II as well as CD86, and be eliminated from the body due to the generation of anti-donor MSC antibodies [14]. Furthermore, allogeneic MSC could be eliminated by CD8^+^ cytotoxic T lymphocytes [15], whereas transplanted autologous or allogeneic MSC could be eliminated by natural killer (NK) cells.

## The MSC secretome

The limited half-life of transplanted cells and potential tumorigenic and other risks of MSC have further prompted development of acellular therapies. While MSC have the ability to differentiate and can thereby contribute to hepatic epithelial replacement, many other observed effects of MSC can be attributed to paracrine effects that occur as a result of factors that are secreted or released from the cells. Indeed, the therapeutic potential of MSC in liver injury could be primarily exerted through paracrine mechanisms that involve the release of soluble proteins or EV, which constitute the MSC secretome.

The use of the MSC secretome as a therapeutic agent is an attractive option that could avoid several of the limitations of cell-based approaches. Consequently, we have focused this review on the potential therapeutic effects of the MSC secretome as an acellular regenerative and reparative therapy for liver injury and disease. There is a growing recognition of the ability of the MSC secretome to modulate the local immune microenvironment, reduce injury and to promote epithelial repair. Undifferentiated MSC lack the expression of co-stimulatory molecules required to activate T cells. Therefore secretome-mediated paracrine effects may be essential contributors to the observed effects of MSC on modulation of immune cells [16].

**Fig. 1:**
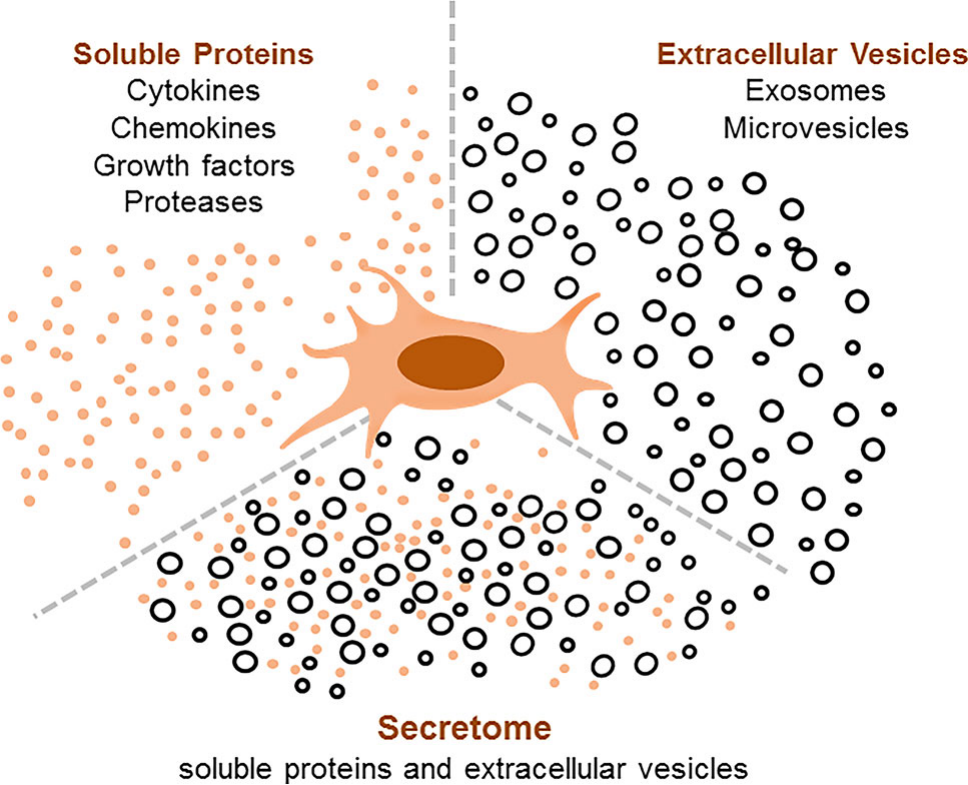
Overview of the MSC secretome. The secretome comprises of soluble proteins and secreted extracellular vesicles. The proteins include biologically active factors such as cytokines (e.g., interleukin 10, and tumor necrosis factor-α), chemokines (e.g., eotaxin-3), and growth factors (e.g., hepatocyte growth factor and transforming growth factor-β isoform 3). The vesicular factors include exosomes and microvesicles

The major constituents of the MSC secretome include a diverse range of soluble proteins and EVs (Fig. 1). Conditioned media (CM) obtained from MSC in culture comprises of a combination of both EVs and protein. The experimental effects observed with the use of CM could result from either or both of these constituents. However, several studies have also reported the effects of EVs that have been isolated and separated out from the secretome. Therapeutically beneficial effects using either CM or separated EV preparations derived from MSC range from promotion of repair to amelioration of injury. In many studies, EV have been isolated from cell culture supernatant or CM using classical centrifugation-based approaches. Although most studies have not directly evaluated for the presence of secreted proteins in EV preparations, the isolation approaches would be expected to eliminate or significantly dilute any secreted protein content. The use of resin-based separation of EV could result in a higher content of secreted proteins which need to be considered when interpreting the results of studies using EV isolated using these approaches.

## Functional effects of the MSC secretome

**Fig. 2:**
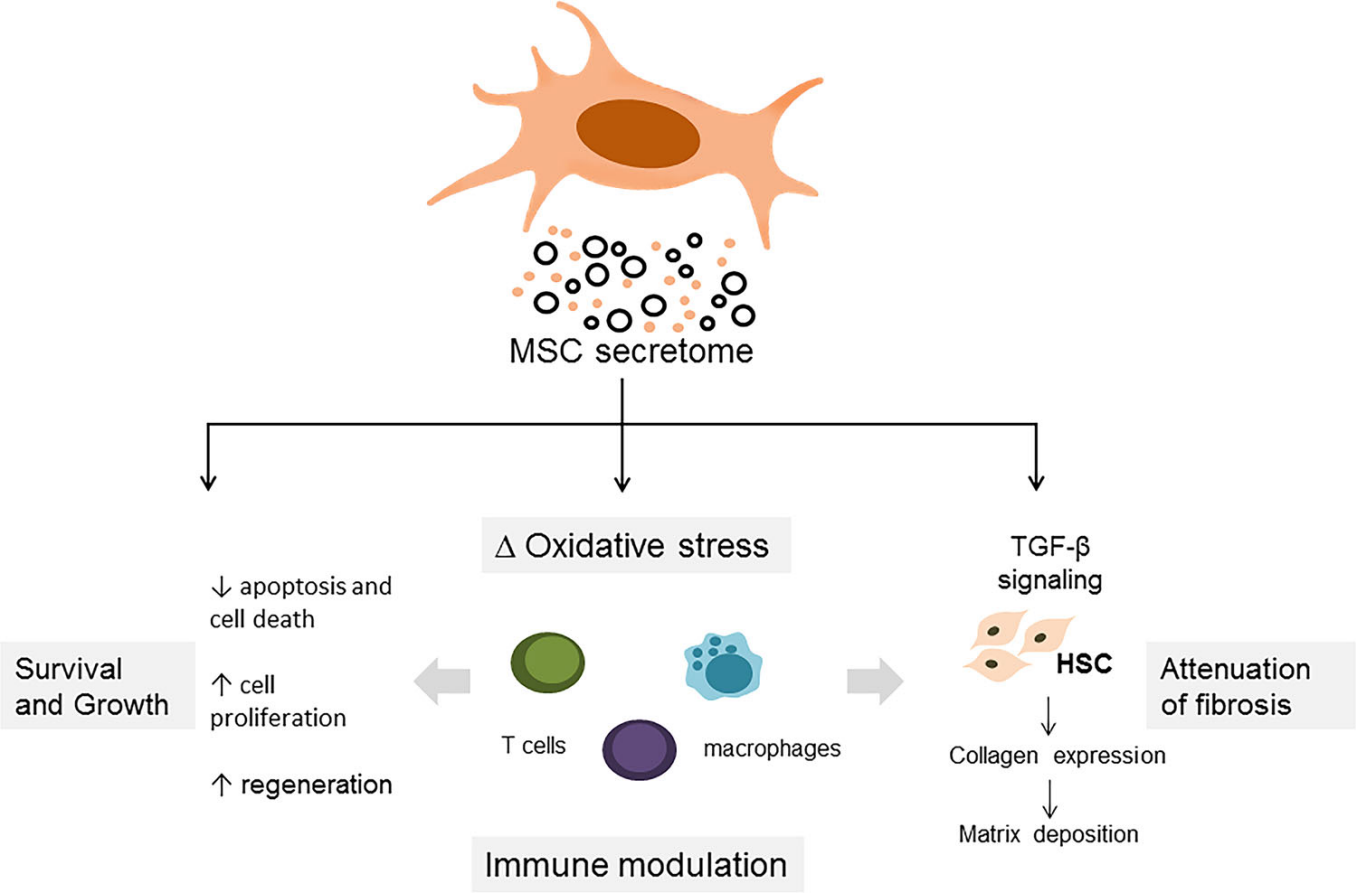
Mechanisms of therapeutic efficacy. The secretome can have a wide range of therapeutically beneficial effects such as immune modulation, amelioration of injury and attenuation of fibrosis. These effects may be mediated by the biological activity of the diverse range of protein, lipid or RNA molecules present within the secretome

The reparative or regenerative properties of the MSC secretome can contribute to immune modulation, amelioration of injury, or reduction of fibrosis (Fig. 2). Soluble proteins such as cytokines and chemokines released by MSC can contribute to several different pathophysiological responses. These can include immunomodulatory effects due to direct or indirect effects on several immune cells or their responses to tissue or cell injury. Growth factors and cytokines within the secretome such as transforming growth factor beta isoform 3 (TGF-β3) [17], hepatocyte growth factor (HGF), IL-10, and tumor necrosis factor-alpha (TNF-α) [18] can modulate cell signaling and processes involved in fibrogenesis and can attenuate liver fibrosis. In addition, the observed paracrine effects of MSC could also result from the EV released from these cells. These EVs comprise of a highly heterogeneous group of vesicles which vary in their size, biogenesis, and content. MSC-derived EV can express MSC surface markers capable of modulating immune responses, as well as specific tetraspannins such as CD63 and CD81 [19]. These EV consist of lipid bilayers enclosing a cargo which can include lipids, proteins, DNA and RNA molecules [20]. Indeed, MSC-derived EV can be selectively enriched with anti-fibrotic [21] and anti-apoptotic [22] proteins or with specific non-coding RNAs [23]. The ability to engineer the production and content of EV offers further opportunities for targeted delivery of specific content for therapeutic applications.

### Immunomodulatory activities

The MSC secretome can favor an immunosuppressive environment through modulating effector cells of both the innate and adaptive immune system. The observed effects of the MSC secretome may involve the direct actions of protein mediators such as IL-10, HGF, TGF- β3, indolamine 2,3 dioxygenase (IDO), and prostaglandin 2 (PGE_2_) and involve modulation of both cellular and adaptive immune responses [17, 24] [25]. For example, MSC-derived PGE_2_ [26] and interleukin (IL)-1ra [27] can induce the polarization of macrophages to adopt an M2 phenotype, which can attenuate inflammation through secretion of IL-10. Likewise, MSC secretion of chemokines, such as macrophage inflammatory protein (MIP) can recruit innate immune cells to the site of injury [16]. The secretion of factors such as nitric oxide (NO) and IDO by MSC in response to inflammatory stimuli can directly impact both the innate and adaptive immune systems. MSC-derived IDO can suppress the proliferation of effector T cells. Furthermore, MSC-CM-derived IDO can contribute to an expansion of CD4^+^CD25^+^ regulatory T cells (Tregs) and attenuate liver injury in α-galactosylceramide (α-GalCer)-injured mice. Activated Tregs secrete IL-10, which can attenuate liver inflammation and reduce natural killer T (NKT) cell-driven effects and hepatotoxicity [24]. Similarly, treatment of thioacetamide (TAA)-injured mice with compact bone-derived MSC-CM reduced the number of infiltrated mononuclear lymphocytes. Similar effects of compact bone-derived MSC-CM were also noted in the CCl_4_-induced model of liver fibrosis, with MSC-CM treatment of CCl_4_-injured mice resulting in increased populations of CD4^+^CD25^+^ Tregs and splenic Cd11b^+^ F4/80^+^ macrophages. Notably, in vitro treatment of macrophages with MSC-CM had no effect on their proliferation [28]. This suggests that MSC-CM could work in concert with factors present in the liver microenvironment to ameliorate drug-induced liver damage. Moreover, the immunomodulatory effects of MSC secretome can be altered by pre-conditioning MSC prior to collecting the secretome. As an example, pre-conditioning of adipose stem cells (ASC) with lipopolysaccharide (LPS) could attenuate the reduction in inflammatory cytokines such as TNF-α and IL-6 [29].

The vesicular component of the MSC secretome can also contribute to immune modulation through direct effects on immune cell activities within the liver microenvironment. The therapeutic effects of MSC-EV treatment are mediated, in part, via the upregulation of IL-6 [30], which can activate signal transducer and activator of transcription 3 (STAT3) signaling and downstream expression of cell survival genes such as B cell lymphoma 2 (Bcl2), Bcl-extra large (Bcl-xL) and FLICE inhibitor protein (FLIP) [31]. The cytoprotective effects of IL-6 related to enhanced expression of Bcl2 and Bcl-xL can protect liver parenchyma from Fas-mediated cell death [32].

While the role of macrophages in attenuating liver damage is controversial, macrophage recruitment appears to contribute to amelioration of liver injury by MSC-EV. In lethal hepatic failure induced by D-galactosamine (D-gal)/TNF-α in mice, administration of MSC-EV increased the number of F4/80^+^ macrophages in the liver concomitant with a reduction in the levels of circulating pro-inflammatory cytokines and attenuation of liver inflammation. Furthermore, MSC-EV treatment increased the serum levels of IL-6 and macrophage inflammatory protein-2 (MIP-2) [23]. Similarly, MSC-EV also increased the number of liver-infiltrating and liver-resident F4/80^+^ macrophages in the murine liver after ischemia reperfusion injury [23, 33].

### Amelioration of liver injury

A growing body of experimental evidence supports the effects of MSC-EVs and secreted proteins in protecting against liver injury or in ameliorating the effect of drug-induced liver injury. The cytoprotective effects of MSC-EV may involve survival signaling by activation of IL-6/STAT3 signaling activity. Treatment of acetaminophen (APAP)- and H_2_O_2_-injured hepatocytes with MSC-derived exosomes, a type of EV, caused a dose-dependent increase in Bcl-xL expression. The increased viability of the toxin-injured hepatocytes was accompanied by upregulated expression of IL-6 and several other inflammatory factors [30]. However, the hepatocyte responses to MSC-EV treatment vary with the type of injury. In APAP-induced hepatocyte injury, MSC-EV can enhance the expression of macrophage inflammatory protein 2 (MIP2) whereas following oxidative injury induced by H_2_O_2_, MSC-EVs enhance expression of inducible nitric oxide synthase (iNOS) [30].

Emerging evidence from several studies highlights the contribution of excipient protein and RNA cargo in the MSC-EVs in contributing to amelioration of oxidative injury. In lethal hepatic failure induced in mice by D-gal/TNF-α, systemic intraperitoneal or intravenous administration of either human or murine MSC-EV decreased hepatic necrosis and increased survival [23]. BM-MSC-EVs reduced apoptosis of hepatocytes isolated from D-gal/TNF-α injured mice. Enrichment of the lncRNA, Y-RNA-1 was noted in the hBM-MSC-EVs and contributed to amelioration of actinomycin D/TNF-α-induced hepatocyte apoptosis in vitro by the BM-MSC-EVs [23].

Similarly, human umbilical cord-derived MSC (hucMSC)-CM treatment reduced oxidative stress and increased the viability of CCL_4_- or H_2_O_2_-injured human hepatocytes (L02 cells). A dose-dependent increase in expression of the anti-apoptotic Bcl2, and reduction in phosphorylated nuclear factor kappa-light-chain-enhancer of activated B cells (NFkβ) were observed in hucMSC-EV-treated CCl_4_-injured L02 cells. hucMSC-CM attenuated oxidative stress-induced apoptosis by suppressing the expression of miR143 [34]. Notably, when compared with unenhanced BM-MSC secretome product, exosome-enrichment enhanced anti-oxidant activity in both APAP- and H_2_O_2_-induced HepG2 cells in vitro and in vivo following CCl_4_-injury [35]. Survival was enhanced in CCL_4_-injured mice treated with hucMSC-EV [22]. Enrichment of the anti-oxidant, glutathione peroxidase 1 (GPX1) was noted in hucMSC-EV [22]. Indeed, EVs from MSCs originating from diverse tissue sources reduced serum aminotransferase levels in CCL_4_-induced liver injury models [22, 35].

MSC-EVs can also protect the liver from hypoxia-induced injury or from ischemia–reperfusion injury. Pre-treatment with murine BM-MSC-EVs reduced serum aminotransferase levels and hepatocyte apoptosis in mice during hepatic ischemia reperfusion injury in vivo and also reduced ROS and decreased NFkβ activity in H_2_O_2_-injured hepatocytes in vitro [33]. Likewise exosome-enriched BM-MSC secretome reduced biochemical markers of liver injury such as serum AST, ALT and bilirubin following ischemia–reperfusion injury in rats. Similar results were observed with the treatment of CCL_4_-injured rats with exosome-enriched BM-MSC secretome [35] (Tables 1 and 2).

**Table 1:** Effects of MSC-CM or MSC secretome in experimental models of liver injury

Source		Injury model	Effect(s)	Mechanism(s)	References
Human liver	IP injection of MSC-CM at the time of injury	Partial hepatectomy	Increased hepatocyte proliferation	Upregulated TNF-α, HGF, TGF-β, IL-1RA, and IL-10	[2]
Umbilical cord	IP injection of undifferentiated or hepatocyte-like MSC secretome	CCl_4_- and TAA-induced liver fibrosis	Reduced number of activated α-SMA^+^ HSC Reduced collagen deposition	Decreased TGF-β signaling	[21]
Human bone marrow	IV injection of MSC-CM	D-Gal-induced liver failure	Decreased hepatocyte apoptosis Reduced serum AST and ALT levels	Increased circulating IL-10 reduced serum TNF- α, IL-6, IL-1ra and attenuated CD45^+^ leukocyte infiltration	[38]
Murine bone marrow	IV injection of MSC-CM	α-GalCer-induced acute liver failure	Reduced serum AST & ALT levels Expanded CD4^+^CD25^+^ T cell infiltration and reduced NKT cell-mediated hepatotoxicity	Suppressed Teff cell proliferation	[24]
Human umbilical cord	MSC- CM	In vitro H_2_O_2_-induced hepatocyte injury	Increased hepatocyte viability	Modulated Bax and Bcl-2 expression	[34]
Murine compact bone	IV injection of MSC-CM	TAA-induced acute liver failure and CCl_4_-induced chronic liver fibrosis	Reduced collagen deposition and α-SMA^+^ cells induced apoptosis of activated HSC in the livers of CCl_4_-injured mice Reduced hepatocyte apoptosis Increased cell proliferation	Reduced hepatic leukocyte infiltration decreased CD11b^+^F4/80^+^ macrophage and Th-17 Induced the expansion of spleen-derived CD4^+^ CD25^+^ Tregs in CCl_4_-injured mice	[28]
Human adipose tissue	MSC- CM (normoxia or hypoxia pre-conditioned)	None	Increased hepatocyte viability (H-CM) Enhanced glycogen and ICG uptake by hepatocytes		[47]
Human umbilical cord	MSC Co-culture	CCl_4_-injured murine hepatocytes	Increased hepatocyte viability Increased albumin production Increased number of proliferating hepatocytes		[37]
Human adipose tissue	IV injection of ASC-CM (Untreated and LPS-primed)	Partial hepatectomy	Increased number of proliferating cells Accelerated liver regeneration Reduced serum transaminase levels	Decreased serum TNF-α and IL-6 levels Increased hepatic expression of p-STAT3 and PCNA	[29]

*α-GalCer* galactosylceramine, *α-SMA* alpha-smooth muscle actin, *ALT* alanine aminotransferase, *AR* adrenergic receptor, *AST* aspartate aminotransferase, *BAX* Bcl2-associated X protein, *Bcl-2* B cell lymphoma 2, *BMF* Bcl2 modifying protein, *CCl*_*4*_ carbon tetrachloride, *CM* conditioned media, *D-gal*d-galactosamine, *EV* extracellular vesicles, *Ex* exosomes, *H* hypoxia, *H*_*2*_*O*_*2*_ hydrogen peroxide, *HB-EGF* heparin-binding EGF-like growth factor, *hBM-MSC* human bone marrow-derived MSC, *HGF* hepatocyte growth factor, *hpucMSC* hepatocyte-like umbilical cord-derived MSC, *HSC* hepatic stellate cells, *hucMSC* human umbilical cord-derived MSC, *ICG* indocyanine green, *IDO* indolamine 2,3 dioxygenase, *IL* interleukin, *IP* intraperitoneal, *IV* intravenous, *LPS* lipopolysaccharide, *N* normoxia, *NKT* natural killer T cells, *OSM* oncostatin M, *PCNA* proliferating cell nuclear antigen, *p-STAT3* phosphorylated signal transducer and activator of transcription 3, *ROS* reactive oxygen species, *SCF* stem cell factor, *SITR1* siturin 1, *SMAD* mothers against decapentaplegic homolog, *SOCS3* suppressor of cytokine signaling, *TAA* thioacetamide, *Teff* effector T cells, *TGF-β* transforming growth factor beta, *TGFRB1* transforming growth factor beta receptor 1, *Th* T-helper cell, *TIMP* tissue inhibitor of metalloproteinases, *TNF-α* tumor necrosis factor-alpha, *Tregs* regulatory T cells, *ucMSC* umbilical cord-derived MSC

**Table 2:** Effects of MSC-EV in experimental models of liver injury

Source		Injury model	Effect(s)	Mechanism(s)	References
Human liver	IV injection of HLSC-MV	Partial hepatectomy	Increased hepatocyte proliferation Reduced apoptosis	Upregulated hepatic expression of cyclin A1	[3]
Human umbilical cord	Intrahepatic injection of MSC-Ex	CCl_4_-induced acute liver injury	Inhibited hepatocyte apoptosis Reduced collagen-1 and -3 expression Reduced the serum levels of HA	Suppressed TGF-β signaling and inhibited EMT	[36]
Human umbilical cord	IV injection of MSC-Ex	CCl_4_-induced liver failure	Increased cell viability	Reduced levels of ROS Upregulated Bcl2 expression	[22]
Human and murine bone marrow	IP and IV injection of MSC-EV	D-gal/TNF-α-induced lethal hepatic failure	Reduced apoptosis Increased survival	Attenuated inflammation Increased macrophages Transfer of Y-RNA-1 within EV	[23]
Murine bone marrow	IV injection of MSC-EV	Hepatic ischemia–reperfusion injury	Reduced apoptosis	Increased Nlrp12 and CXCL1 Increased number of macrophages Altered NFkβ activity and decreased cytokine and growth factors Reduced ROS	[33]
Human huE59.E1-	Intrasplenic injection of MSC-EV	CCl_4_-induced acute liver injury In vitro APAP- and H_2_O_2_-induced hepatocyte injury	Decreased apoptosis Increased cell viability	Upregulated cyclin D, NFkβ and cyclin E expression reduced caspase 3 activity, restored Bcl-xL expression and increased the amount of activated STAT3 Increased expression of immune mediators: TNF-α, IL-6, iNOS, COX-2 and MIP-2	[30]
Rat bone marrow	Intrahepatic injection of MSC-exosome-enriched fraction	In vitro H_2_O_2_- and APAP-induced HepG2 injury CCL_4_-induced acute liver injury and IRI	Increased hepatic regeneration Reduced serum AST, ALT, and bilirubin levels Protected HepG2 cells from toxin-induced death Promoted hepatocyte proliferation	Significantly reduced ROS levels and LDH activity in toxin-injured HepG2 cells Reduced the number of 8-OHdG^+^ hepatocytes in CCL_4_-injured animals	[35]
Adipose tissue	IV injection of MSC secretome (1%, 5%, 10%, and 21% pO_2_*)*	In vitro IRI Partial hepatectomy	Reduced serum IL-6 and TNF-α levels Reduced serum transaminases Accelerated liver regeneration Increased the hepatocyte proliferation	Increased p-STAT3 and PCNA expression Decreased hepatic expression of SOCS3 and increased SIRT1 Increase in survival genes (e.g., Bcl-xL and Mcl-1)	[41]

*α-SMA* alpha-smooth muscle actin, *ALT* alanine aminotransferase, *AST* aspartate aminotransferase, *BAX* Bcl2-associated X protein, *Bcl-2* B cell lymphoma 2, *Bcl-xL* B cell lymphoma-extra large, *BMF* Bcl2 modifying protein, *CCl*_*4*_ carbon tetrachloride, *CM* conditioned media, *CXCL1* chemokine (C-X-C motif) ligand 1, *d-gal*d-galactosamine, *EMT* epithelial to mesenchymal transition, *EV* extracellular vesicles, *Ex* exosomes, *H*_*2*_*O*_*2*_ hydrogen peroxide, *hBM-MSC* human bone marrow-derived MSC, *HGF* hepatocyte growth factor, *HLSC* human resident liver stem cells, *hpucMSC* hepatocyte-like umbilical cord-derived MSC, *hucMSC* human umbilical cord-derived MSC, *huE59.E1* fetal tissue-derived MSC, *IFN-γ* interferon gamma, *IL* interleukin, *IP* intraperitoneal, *iNOS* inducible nitric oxide synthase, *IRI* ischemia reperfusion injury, *IV* intravenous, *MIP2* macrophage inflammatory protein 2, *MV* microvesicles, *NFkβ* nuclear factor kappa-light-chain-enhancer of activated B cells, *Nlrp12* NLR family pyrin domain containing 12, *PCNA* proliferating cell nuclear antigen, *pO*_*2*_ partial pressure of oxygen, *STAT3* signal transducer and activator of transcription 3, *ROS* reactive oxygen species, *SMAD* mothers against decapentaplegic homelog, *SOCS3* suppressor of cytokine signaling, *STAT3* signal transducer and activator of transcription 3, *TAA* thioacetamide, *TGF-β* transforming growth factor beta, *TNF-α* tumor necrosis factor-alpha, *ucMSC* umbilical cord-derived MSC, *VEGF* vascular endothelial growth factor

### Anti-fibrotic effects

The use of MSC secretome has been shown to have anti-fibrotic effects. In one study, MSC secretome reduced fibrosis induced by TAA. Secretome was obtained by ultrafiltration of CM from cultures of umbilical cord (uc)-derived MSC that were either undifferentiated or had been primed to differentiate into hepatocyte-like cells (hpucMSC). The reduction in fibrosis was associated with a decrease in extracellular matrix (ECM) deposition, and accompanied by a reduction in the number of alpha-smooth muscle actin (α-SMA)-positive hepatic stellate cells, and down regulation of pro-fibrogenic genes, TGF-β and several downstream targets (mothers against decapentaplegic homolog (Smad)-2, -3, -4, and -6). The secretome product was enriched in the milk factor globule EGF 8 (MFGE8), an anti-fibrotic protein that is reduced in fibrotic or cirrhotic livers [21]. The secretome products did not induce apoptosis in LX2 hepatic stellate cells but inhibited TGF-β-induced HSC activation. In contrast, compact bone-derived MSC-CM can promote apoptosis of LX2 cells in vitro [28]. These observations suggest that the secretome from different cellular sources could have divergent effects on the target cells. They indicate a need to correlate functional effects in target cells and tissues with cell type-specific secretome production and composition.

Anti-fibrotic effects have also been observed with the use of isolated EV from MSC. Intrahepatic injection of hucMSC-EVs decreased liver fibrosis, reduced apoptosis and mitigated liver damage induced by CCl_4_ in mice. Anti-fibrotic changes were associated with a decrease in TGF-β signaling and a reduction in collagen-1 and collagen-3 expression, with the greatest effects observed at 3 weeks post treatment. An effect of MSC-EV on epithelial to mesenchymal transition (EMT) was suggested by a significant decrease in N-cadherin^+^ and vimentin^+^ and increase in E-cadherin^+^ liver cells. Exposure of HL7702 human epithelioid liver cells to TGF-β for 3 days induces their trans-differentiation into fibroblasts. Subsequent treatment with hucMSC-derived exosomes (Ex) reduced the mRNA expression of EMT markers, N-cadherin and Twist [36]. Taken together, these results suggest that MSC-EV can protect and restore the functional activity of liver parenchyma in surgical and drug-induced models of liver injury.

### Liver regeneration

Several studies have reported the effects of MSC and the MSC secretome on enhancing cell proliferation and liver regeneration [35, 37]. MSC-CM treatment reduced hepatocyte apoptosis and improved the survival of D-gal-injured rats. Interestingly, MSC-CM treatment upregulated the expression of several genes that are implicated in liver regeneration, such as oncostatin M, adrenergic receptor-1 and stem cell factor [38]. Co-culture of hucMSC increased viability, functional activity, and proliferation of murine hepatocytes following CCl_4_ injury in vitro [37]. The administration of MSC secretome can also promote liver regeneration in vivo following partial hepatectomy in mice. The vesicular fraction of the secretome may encompass part or all of the beneficial properties, as treatment with exosome-enriched BM-MSC secretome was shown to increase the rate of liver regeneration following ischemia–reperfusion injury in partially hepatectomized rats [35].

Pre-conditioning the MSC prior to collection of the secretome product can enhance desired properties. Acceleration of the regenerative response, as well as beneficial effects on reducing hepatic injury or increasing hepatocyte proliferation were enhanced with the use of secretome obtained from ASC that underwent LPS pre-conditioning compared with unstimulated ASC [29, 39, 40]. Similarly, hypoxia pre-conditioning of ASC engendered a secretome product that promoted liver regeneration, with the greatest benefit observed with secretome obtained from MSC exposed to 1% partial pressure of oxygen (pO_2_) [41]. Hepatocyte proliferation in hypoxia-primed ASC-CM-treated mice was increased following partial hepatectomy and was associated with a reduced suppression of cytokine signaling 3 (SOCS3) expression, enhanced STAT3 signaling and increased HGF, VEGF, and Bcl-XL expression [42]. As these studies indicate, the regenerative capabilities and hence the therapeutically beneficial properties of stem cell-derived secretome products could be enhanced by pre-conditioning protocols. Further work to define the optimal protocols required for specific desirable properties will be necessary and required prior to translational application of these strategies.

## MSC secretome as a therapeutic agent

**Fig. 3:**
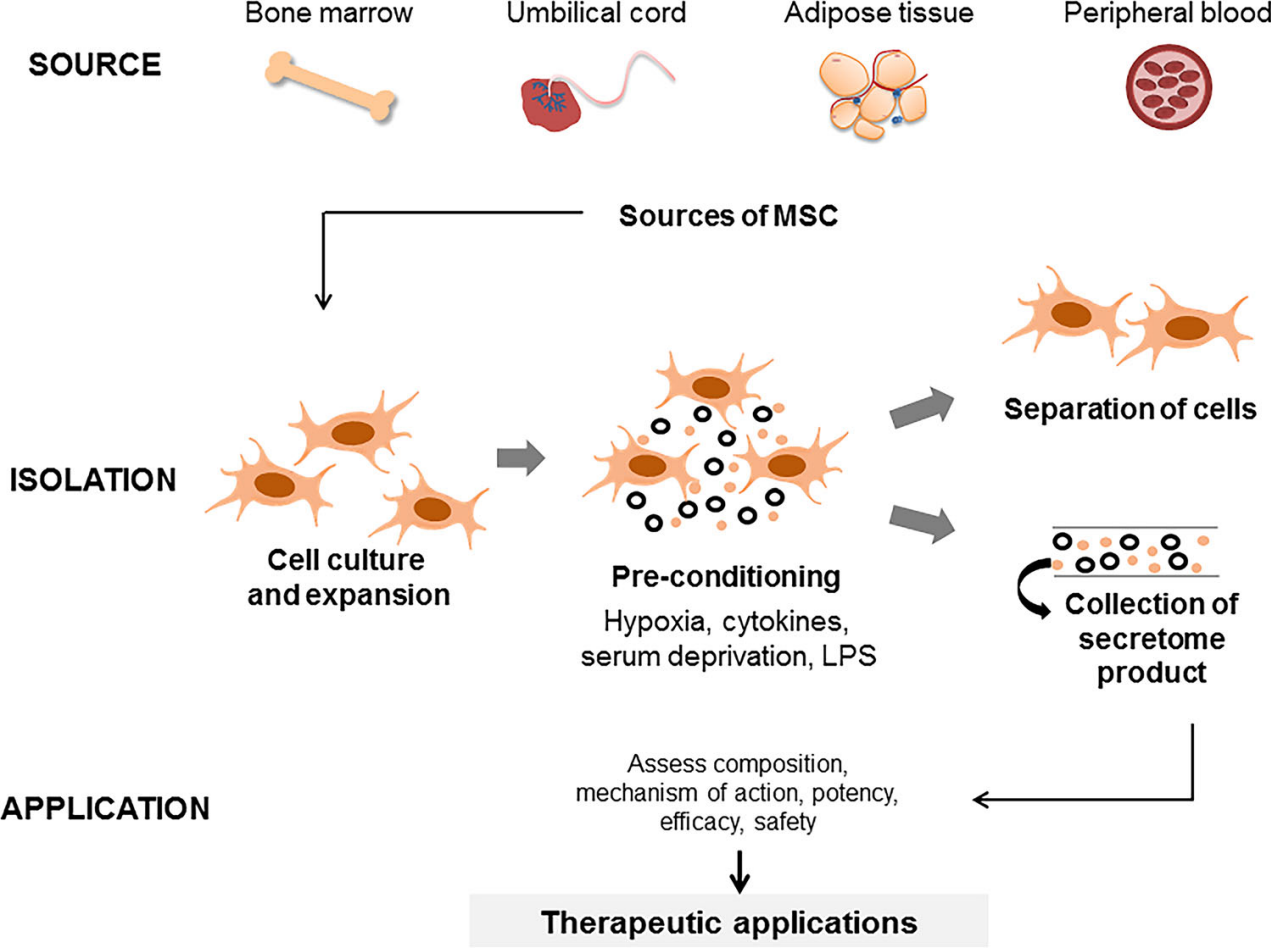
Approaches to developing an MSC secretome-based therapeutic agents. An MSC secretome-based therapeutic product can be generated from autologous stem cells obtained from common sources such as bone marrow, or adipose tissue. The cells are expanded in culture, followed by conditioning to enhance release of soluble proteins or EV and resulting in an enhanced secretome product. The conditioned media is subsequently collected and further isolation procedures to remove the cellular components, such as using ultracentrifugation or tangential flow filtration, can be performed to isolate the acellular secretome product

The MSC secretome has several advantages for further development as a therapeutic agent even though the precise mediators of the cytoprotective effects are not known. An approach to the development of an MSC secretome-based therapeutic product is illustrated in Fig. 3. While there is growing interest in the use of isolated MSC-EV preparations for therapeutic purposes, the use of a secretome product that contains both EV and soluble proteins also provides an attractive physiological approach to maintaining tissue homeostasis in the setting of injury. Given the diverse range of proteins that are secreted and the heterogeneity of vesicles that are released from MSC, it is unlikely that a unique factor can be isolated. However, a secretome therapeutic product could allow for the enrichment of therapeutic factors and thereby maximizes the therapeutic effect.

The protein and vesicular content of the MSC secretome could be manipulated in several ways to improve desired functional effects. Several approaches have been described to condition stem cells for the manipulation of secretome or EV contents. These include culture conditions such as modulation of oxygen tension, variations in matrix, serum deprivation, fluid shear or compression, or culture in 3-dimensional cultures. Hypoxic pre-conditioning of MSCs, for example, can enhance the angiogenic potential of the secretome product [43]. Other approaches to condition stem cells include the use of pro-inflammatory stimuli such as cytokines or LPS, as noted above, or the use of pro-differentiation stimuli. Hypoxia conditioning can increase the release of chemokines and several immune mediators such as IL-6, IL-15, and IL-1b [44]. Cytokine priming of BM-MSC using TNF-α and IFN-ϒ can reduce the secretion of cytokines such as IL-10, IL-5, IL-6, and IL-13, and enrich expression of cyclooxygenase 2 (COX2) [45]. Induction of differentiation can also alter the secretome. The secretome product from MSC of umbilical cord origin that were differentiated into hepatocyte-like cells contains higher amounts of the glycoprotein, MFGE8 and has a superior anti-fibrotic effect [21]. Finally, the underlying source of MSC may also be relevant. Thus, MFEG8 is most enriched in BM-, uc- and SHED (teeth)-derived MSC secretomes, and further enhanced with hepatocyte differentiation of these cells. In contrast, the embryonic stem cell-derived secretome does not contain MFGE8 [21].

These observations not only highlight the heterogeneous nature of the protein and EV content, and functional variability of the MSC secretome but provide opportunities for bespoke functional applications based on detailed knowledge of the underlying cell-specific behavior, state of differentiation and response to the external milieu. They emphasize an essential need for standardized approaches to isolation, conditioning, and characterization of potency of secretome products for therapeutic applications. These would facilitate the performance and interpretation of clinical trials to determine the safety and efficacy of secretome-based therapy.

## Future perspectives

The MSC secretome is an attractive emerging option for therapeutic applications as an acellular regenerative or reparative therapy for liver injury and disease. MSCs are present in almost all post-natal tissues, including adipose tissue, bone marrow and umbilical cord [46]*.* Although there are several clinical trials to evaluate the use of cell-based therapies, to date there have been no studies focused on the use of acellular secretome-based products for liver diseases, despite their advantages over cell-based approaches. The availability of autologous MSC as a source of the MSC secretome is feasible. Further efforts to optimize the isolation and therapeutic efficacy of a potent MSC secretome product and to define appropriate indications for therapeutic applications are warranted. Based on the results of the available pre-clinical studies, the MSC secretome should be further explored and translational studies to evaluate the clinical use are justified. We hope that this concise review will stimulate and encourage such efforts towards harnessing the therapeutic potential of the MSC secretome.

## Abbreviations

α-GalCer: Galactosylceramide
α-SMA: Alpha-smooth muscle actin
APAP: Acetaminophen
Bcl-2: B cell lymphoma 2
Bcl-xL: B cell lymphoma-extra large
CCI_4_: Carbon tetrachloride
CM: Conditioned media
COX2: Cyclooxygenase 2
d-gal: d-Galactosamine
ECM: Extracellular matrix
EMT: Epithelial to mesenchymal transition
EV: Extracellular vesicles
Ex: Exosomes
FLIP: FLICE inhibitor protein
GPX1: Glutathione peroxidase 1
H_2_O_2_: Hydrogen peroxide
hBM-MSC: Human bone marrow-derived MSC
HGF: Hepatocyte growth factor
hpucMSC: Hepatocyte-like umbilical cord-derived MSC
hucMSC: Human umbilical cord-derived MSC
IDO: Indolamine 2,3 dioxygenase
IFN-γ: Interferon gamma
IL: Interleukin
iNOS: Inducible nitric oxide synthase
LPS: Lipopolysaccharide
mBM-MSC: Murine bone marrow-derived MSC
MFGE8: Milk factor globule EGF 8
MIP2: Macrophage inflammatory protein 2
miR: Micro-RNA
MMP: Matrix metalloproteases
MSC: Mesenchymal stem cells
ncRNA: Non-coding RNA
NFkβ: Nuclear factor kappa-light-chain-enhancer of activated B cells
NK: Natural killer cells
NKT: Natural killer T cells
NO: Nitric oxide
ROS: Reactive oxygen species
SMAD: Mothers against decapentaplegic homolog
SOCS3: Suppression of cytokine signaling 3
STAT3: Signal transducer and activator of transcription 3
TAA: Thioacetamide
TGF-β: Transforming growth factor beta
TNF-α: Tumor necrosis factor-alpha
Tregs: Regulatory T cells
